# Analysis of prognostic factors for R1/R2 resection in patients with hilar cholangiocarcinoma

**DOI:** 10.1186/s12893-026-03776-5

**Published:** 2026-05-06

**Authors:** Zepu Wang, Chuncheng Wang, Meijian Yang, Dan Lv, Yanhui Peng

**Affiliations:** 1 Department of hepatobiliary and pancreatic surgery II, Hebei General Hospital, 348 Heping West Road, Shijiazhuang City, Hebei Province 050051 China; 2 Ultrasound department, Hebei General Hospital, Shijiazhuang City, Hebei Province China; 3 Department of oncology II, Hebei General Hospital, Shijiazhuang City, Hebei Province China

**Keywords:** Hilar cholangiocarcinoma, R1/R2 resection, Nomogram model, Prognostic

## Abstract

**Objective:**

To identify prognostic factors and develop a predictive tool for patients with hilar cholangiocarcinoma (HCCA) undergoing R1 or R2 resection, thereby informing patient selection and individualized treatment decisions.

**Methods:**

A retrospective analysis was conducted of HCCA patients who underwent R1 or R2 resection at a single center. Independent prognostic factors were identified using Cox regression analysis, and a predictive nomogram was constructed using R software.

**Results:**

Multivariate analysis identified four independent prognostic factors: surgical margin status (*P* = 0.002), tumor differentiation grade (*P* = 0.030), vascular invasion (*P* < 0.001), and adjuvant therapy (*P* = 0.023). The nomogram based on these factors demonstrated favorable discriminatory ability, with a C-index of 0.780. Time-dependent receiver operating characteristic (ROC) analysis yielded areas under the curve (AUC) of 0.904 (95% confidence interval [CI]: 0.831–0.966) and 0.822 (95% CI: 0.736–0.897) for predicting 1-year and 2-year survival, respectively. Patients stratified into high-risk and low-risk groups by the nomogram showed significantly different survival outcomes (1-year survival: 44% vs. 92.5%; 2-year survival: 20% vs. 52.8%).

**Conclusion:**

The developed nomogram effectively predicts prognosis following R1 or R2 resection for HCCA, demonstrating good discrimination and short-term predictive accuracy. It serves as a useful tool for postoperative risk stratification and personalized management planning.

**Supplementary Information:**

The online version contains supplementary material available at 10.1186/s12893-026-03776-5.

## Introduction

Hilar cholangiocarcinoma (HCCA) is a malignant tumor arising from the mucosal epithelium of the bile ducts at the hepatic hilum, specifically between the origins of the secondary hepatic ducts and above the cystic duct insertion. It accounts for 50–70% of all cholangiocarcinomas [1]. Pathologically, HCCA is predominantly an adenocarcinoma that induces marked fibrous stromal hyperplasia, typically manifesting as a sclerosing stricture or mass [2]. Its location within the porta hepatis is anatomically complex, adjacent to the confluence of the bile ducts. The tumor frequently encases the portal vein and hepatic artery, leading to early vascular and perineural invasion. A propensity for infiltration along neural and lymphatic pathways results in regional lymph node metastasis, which is present in approximately 40% of patients at diagnosis [3–4]. Biologically, HCCA is characterized by submucosal spread and nonspecific early symptoms. Consequently, about 70% of patients present with advanced disease, and only 30–40% are candidates for curative radical (R0) resection [5].

For patients ineligible for R0 resection, R1 or R2 resection may offer a significant survival and quality-of-life advantage over palliative drainage alone. Reported 1-year and 2-year survival rates after R1/R2 resection are 71% and 19%, respectively, compared with 48% and 12% for drainage alone [6]. However, the benefit is not universal; some patients may not derive a significant survival advantage from the added surgical trauma [7]. Preliminary observations from our center suggest that the benefit of R1/R2 resection may vary with disease stage (unpublished data). Therefore, a precise assessment of prognostic factors in this specific patient cohort is crucial.

The present study aimed to address this need by performing a retrospective analysis of clinical data from HCCA patients who underwent R1 or R2 resection. Our objectives were to identify factors influencing prognosis in this setting and to develop a framework for selecting patients most likely to benefit from this approach, thereby informing individualized treatment strategies.

## Materials and methods

### Research population

We retrospectively reviewed the clinical data of patients with HCCA who underwent R1 or R2 resection at Hebei Provincial People’s Hospital between July 2016 and July 2023. Patients were included if they were aged ≥ 18 years and had pathologically confirmed HCCA with R1 or R2 resection margins. Exclusion criteria were as follows: (1) concurrent or prior malignancy in another organ system; (2) pregnancy or lactation; (3) pathologically confirmed R0 resection; or (4) incomplete clinical or pathological records. Based on these criteria, 103 patients were included in the final cohort.

### Definition of the study cohort

This study focused on HCCA patients who underwent surgery with histopathologically confirmed residual tumor, classified as R1 (microscopic) or R2 (macroscopic) resection. Although R1 resection is often termed “radical resection with positive margins” in surgical intent, both R1 and R2 indicate incomplete tumor clearance and are associated with a similarly unfavorable prognosis compared with R0 resection [8–9]. For the purpose of developing a prognostic model for patients with residual disease, we combined R1 and R2 resections into a single analytical cohort.

### Research methods

The following patient data were collected by reviewing medical records: sex, age, Eastern Cooperative Oncology Group (ECOG) performance status, preoperative total bilirubin (TBil), carbohydrate antigen 19 − 9 (CA19-9), tumor diameter, resection margin status, tumor differentiation grade, lymph node metastasis status, vascular invasion status, and the occurrence of postoperative complications.

Patient follow-up was conducted through telephone contact, outpatient visits, and inpatient re-examinations to ascertain survival status and the subsequent need for adjuvant therapy. Follow-up was performed monthly until the study cutoff date of July 31, 2025, or until patient death.

Adjuvant therapy was defined as any postoperative systemic treatment initiated within 8 weeks of surgery. During the study period, this consisted almost exclusively of gemcitabine-based chemotherapy, as radiotherapy was used in fewer than 5% of cases and immunotherapy was not routinely available at our institution during this timeframe. Therefore, the term “adjuvant therapy” in this study primarily reflects the effect of chemotherapy.

### Sample size

Given the low incidence of HCCA, conducting studies with large cohorts at a single institution is inherently challenging. The present analysis included 103 patients who underwent R1 or R2 resection, a sample size comparable to that of several published prognostic studies in this field [10–12]. The final nomogram incorporated four predictor variables and was developed based on 65 observed events (deaths) at the primary endpoint. The resulting events per variable (EPV) ratio of 16.3 (65/4) exceeds the widely recommended threshold of 10, which helps ensure the model’s robustness against overfitting.

### Statistical methods

Data were analyzed using SPSS version 21.0 and R software. Continuous variables were dichotomized using clinical thresholds based on previous studies [13] and institutional clinical practice for Kaplan–Meier and log-rank analyses. Variable selection was performed using least absolute shrinkage and selection operator (LASSO) Cox regression. Variables selected by LASSO were then simultaneously entered into a multivariable Cox proportional hazards model to identify independent prognostic factors. Multicollinearity was assessed using variance inflation factors (VIF < 5). The proportional hazards assumption was verified using Schoenfeld residual tests (all *p* > 0.05). A nomogram was constructed based on the final model. Discrimination was evaluated using the C-index and time-dependent area under the curve (AUC). Internal validation was performed using 1000 bootstrap samples for calibration and bias-corrected C-index. Categorical variables were compared using the chi-square test or Fisher’s exact test, as appropriate. Survival curves were compared using the log-rank test. A two-tailed *P* < 0.05 was considered statistically significant.

## Results

### Univariate analysis of factors affecting prognosis

The clinical characteristics of the 103 patients with HCCA who underwent R1 or R2 resection are presented in Table 1. Univariate analysis of 13 prognostic factors revealed that the following variables were significantly associated with prognosis: preoperative CA19-9 level (*P* = 0.039), resection margin status (*P* < 0.001), tumor differentiation grade (*P* = 0.014), lymph node metastasis (*P* = 0.012), vascular invasion (*P* < 0.001), and adjuvant therapy (*P* < 0.001) (Table 1).

**Table 1:** Univariate analysis of prognostic factors following R1/R2 resection for HCCA

Clinical factor	Number of case	2-Year Survival,n (%)	χ²	*P*
Gender				
Male	63	24(38.10)	0.085	0.770
Female	40	14(35.00)		
Age (years)				
≤65	40	18(45.00)	1.804	0.179
＞65	63	20(31.75)		
Preoperative TBil (umol/L)				
≤171	53	22(41.51)	1.053	0.305
＞171	50	16(32.00)		
Preoperative CA199(U/mL)				
≤200	48	22(45.83)	4.278	0.039
＞200	55	16(29.09)		
Preoperative albumin(g/L)				
≤35	39	12(30.77)	1.096	0.295
＞35	64	26(40.63)		
ECOG score				
≤1	59	23(38.98)	0.921	0.337
＞1	44	15(34.09)		
Resection margin status				
R1	65	34(52.31)	42.634	＜0.001
R2	38	4(10.53)		
Differentiation grade				
Well	25	12(48.00)	6.019	0.014
Moderate	43	18(41.86)		
Poor	35	8(22.86)		
Tumor diameter (cm)				
≤2	56	22(39.29)	0.708	0.400
＞2	47	16(34.04)		
Lymph node metastasis				
Yes	35	9(25.71)	6.354	0.012
No	68	29(42.65)		
Vascular invasion				
Yes	28	2(7.14)	48.198	＜0.001
No	75	36(48.00)		
Adjuvant therapy				
Yes	78	34(43.59)	24.257	＜0.001
No	25	4(16.00)		
Postoperative complications				
Yes	37	11(29.73)	1.350	0.245
No	66	27(40.91)		

### Multivariate analysis of factors affecting prognosis

The six factors that were significant in univariate analysis were entered into the multivariable Cox proportional hazards model. The results demonstrated that vascular invasion (HR = 4.088, 95% CI: 2.269–7.364; *P* < 0.001) and R2 resection margin status (vs. R1, HR = 2.652, 95% CI: 1.448–4.854; *P* = 0.002) were independent risk factors for poorer survival. Conversely, adjuvant therapy (HR = 0.441, 95% CI: 0.218–0.894; *P* = 0.023) and better tumor differentiation (HR = 0.564, 95% CI: 0.336–0.947; *P* = 0.030) were independent protective factors. Lymph node metastasis (HR = 1.107, 95% CI: 0.604–2.030; *P* = 0.742) and preoperative CA19-9 level (HR = 1.050, 95% CI: 0.597–1.846; *P* = 0.865) did not retain independent significance in the multivariable model (Table 2).

**Table 2:** Multivariate analysis of prognostic factors following R1/R2 resection for HCCA

Clinical factor	β	SE	χ²	HR (95% CI)	P value
Preoperative CA199	0.049	0.288	0.029	1.050 (0.597-1.846)	0.865
Resection margin status	0.975	0.308	9.992	2.652 (1.448-4.854)	0.002
Differentiation grade	-0.573	0.264	4.700	0.564 (0.336-0.947)	0.030
Lymph node metastasis	0.102	0.309	0.109	1.107 (0.604-2.030)	0.742
Vascular invasion	1.408	0.300	21.986	4.088 (2.269-7.364)	<0.001
Adjuvant therapy	-0.819	0.361	5.154	0.441 (0.218-0.894)	0.023

### Construction and verification of the nomogram model

**Fig. 1:**
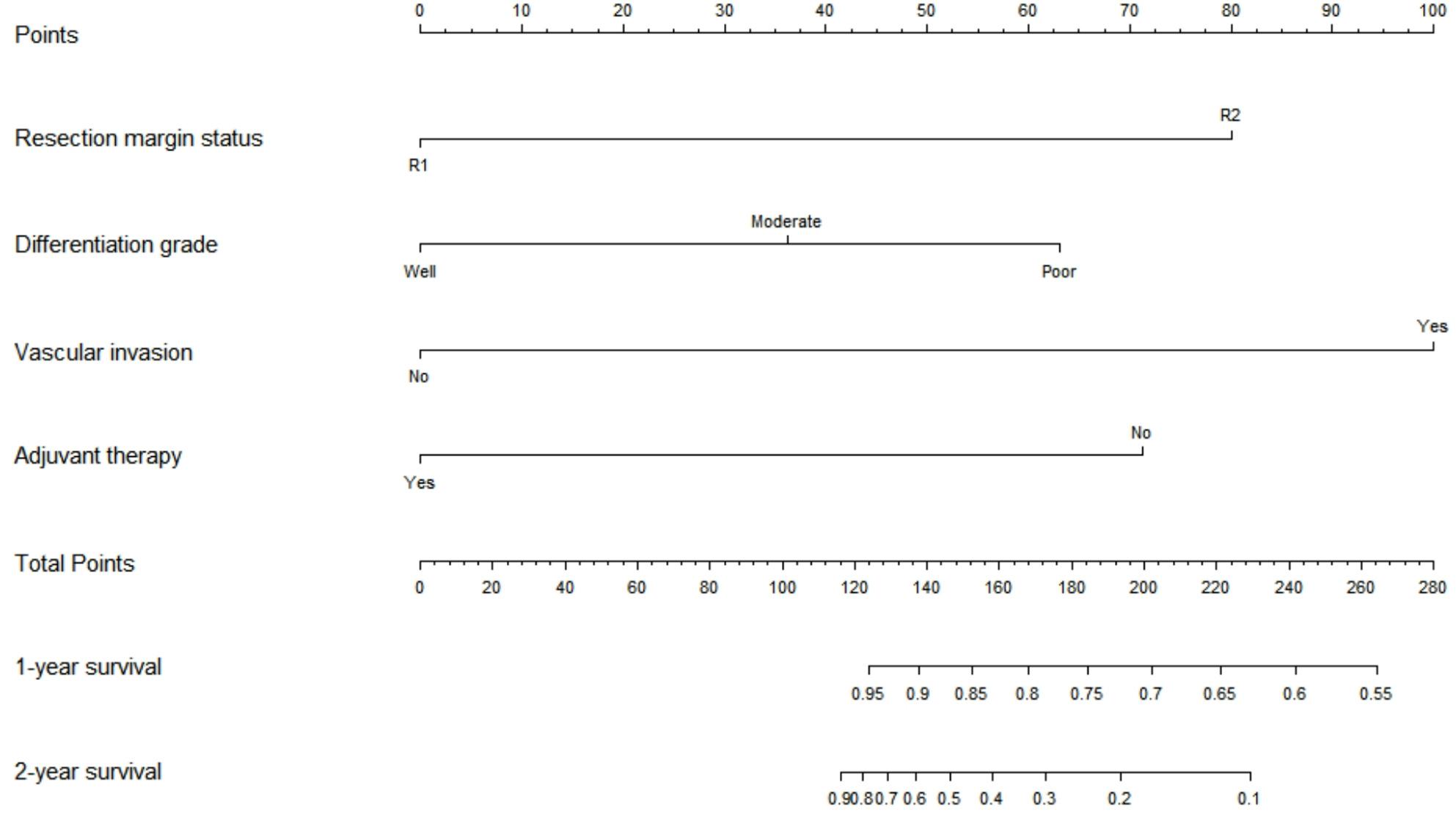
Nomogram model for predicting the prognosis of HCCA patients undergoing R1/R2 resection surgery

**Fig. 2:**
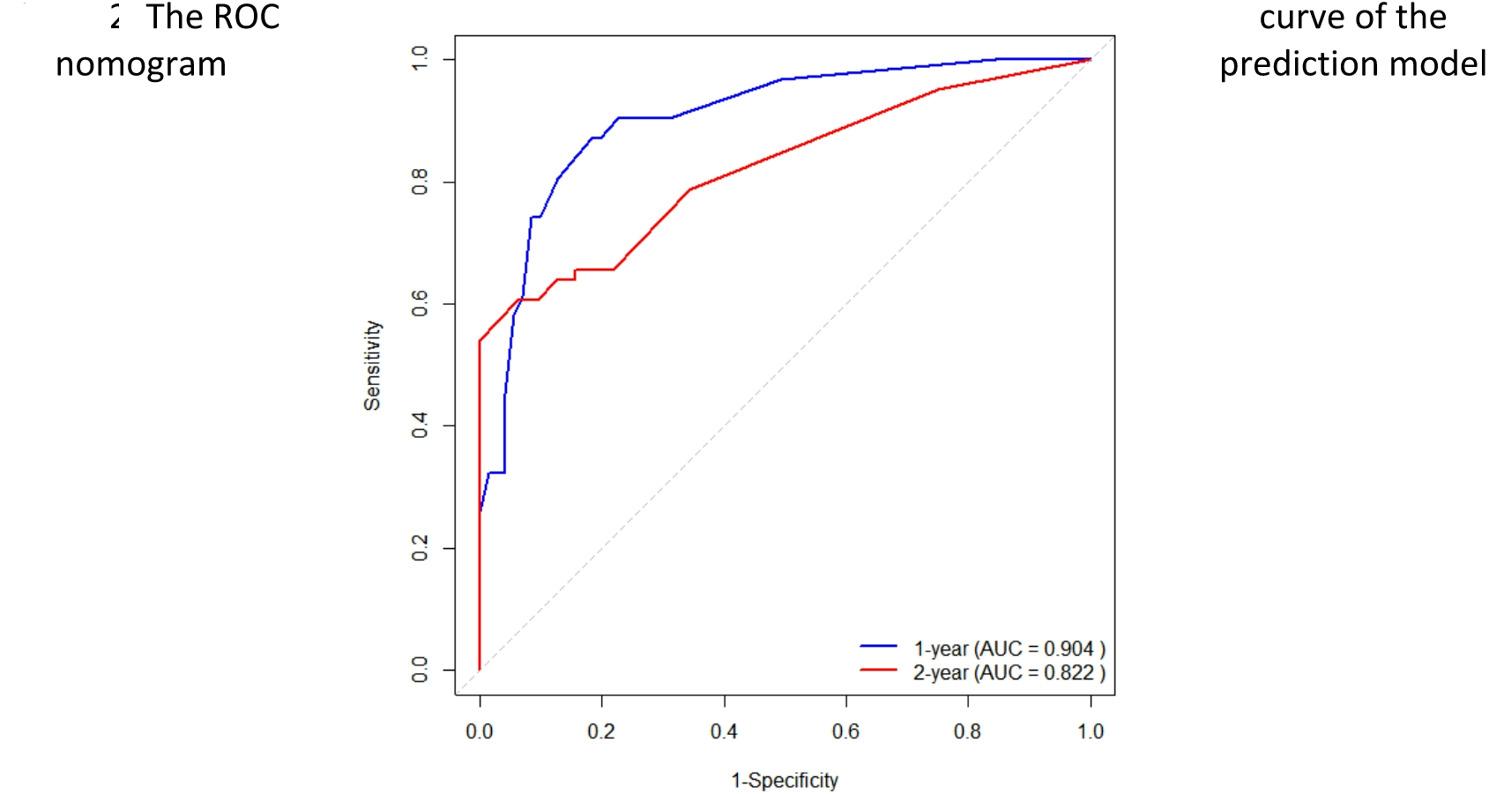
The ROC curve of the nomogram prediction model

**Fig. 3:**
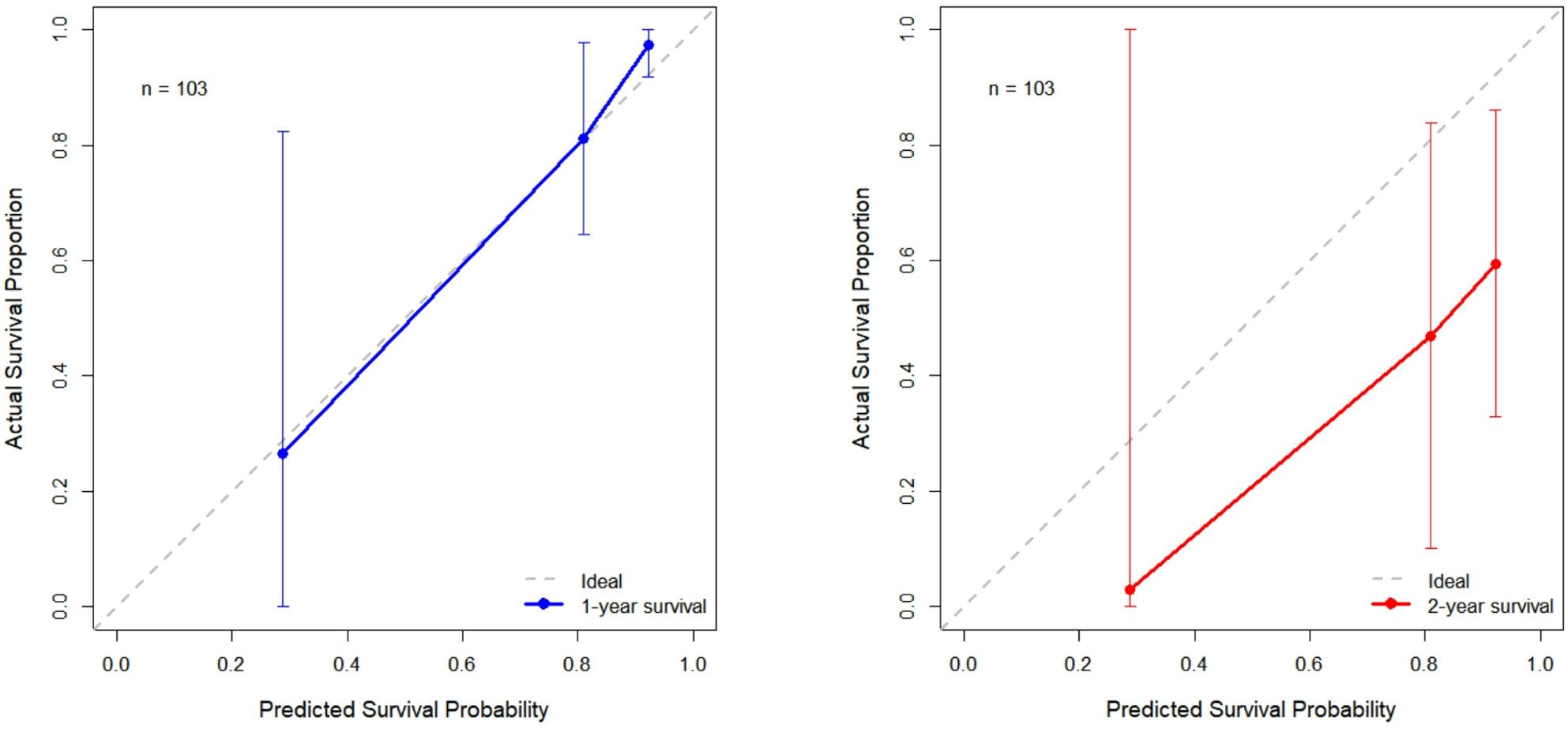
The calibration curve of the nomogram prediction model

A prognostic nomogram for HCCA patients undergoing R1 or R2 resection was constructed using R software based on the results of multivariable Cox regression analysis (Fig. 1). The model demonstrated good discriminative ability, with a C-index of 0.780. To evaluate the model’s performance at different time points, time-dependent receiver operating characteristic (ROC) curve analysis was performed. The areas under the curve (AUC) for 1-year and 2-year survival were 0.904 (95% confidence interval [CI]: 0.831–0.966) and 0.822 (95% CI: 0.736–0.897), respectively (Fig. 2), confirming the model’s excellent predictive ability. Calibration curves were generated to assess predictive accuracy. The predicted 1-year survival probability showed high consistency with the observed survival, whereas the predicted 2-year survival was overestimated (Fig. 3).

### Survival analysis based on nomogram risk stratification

**Fig. 4:**
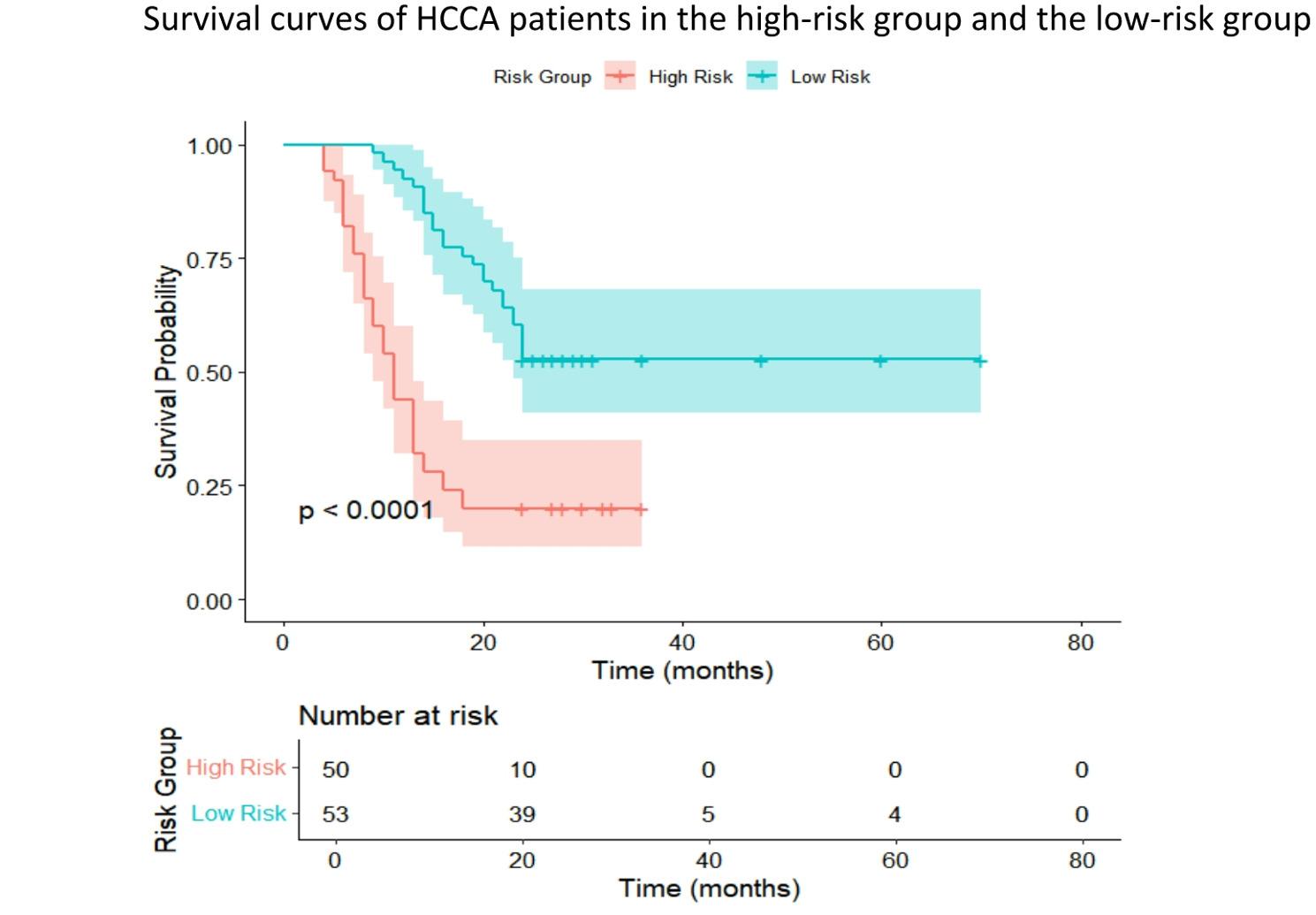
Survival curves of HCCA patients in the high-risk group and the low-risk group

Based on the risk score calculated by the nomogram, all patients were divided into low-risk (*n* = 53) and high-risk (*n* = 50) groups using the median risk score as the cutoff value. Kaplan–Meier survival analysis revealed a significant difference in postoperative survival between the two groups (*P* < 0.001). The median survival time for the high-risk group was 11 months, whereas the median survival for the low-risk group was not reached during the follow-up period. The 1-year and 2-year cumulative survival rates for the high-risk group were 44% and 20%, respectively, which were significantly lower than the corresponding rates for the low-risk group (92.5% and 52.8%, respectively). These findings confirm that the nomogram effectively distinguishes between patient groups with different prognostic outcomes (Fig. 4).

## Discussion

Due to its unique anatomical location and aggressive biology, HCCA remains one of the most challenging entities in hepatobiliary surgery. Radical (R0) surgical resection offers the best chance for long-term survival, with reported 5-year survival rates of approximately 30.1% [14–15]. However, achieving R0 resection is technically demanding, often requiring major hepatectomy, lymphadenectomy, and sometimes vascular resection and reconstruction. Consequently, only 30–40% of patients are candidates for this curative approach [5].

For patients ineligible for R0 resection, the role of cytoreductive (R1/R2) surgery is debated. Evidence suggests that R1/R2 resection can improve survival compared with palliative drainage alone, despite yielding lower survival rates than R0 resection [16–18]. Conversely, other studies indicate that not all patients benefit; the added surgical morbidity may prolong recovery and delay systemic therapy without a significant survival gain [19–20]. This underscores the critical need to accurately identify which patients will derive a true benefit from R1/R2 resection—a key challenge in clinical management.

Although the prognostic factors following R0 resection for HCCA—such as margin status, tumor differentiation, lymph node metastasis, vascular invasion, postoperative complications, and preoperative CA19-9 and albumin levels—are well established [21–22], the determinants of outcome after R1/R2 resection remain less defined. To address this gap, we analyzed a cohort of 103 HCCA patients who underwent R1 or R2 resection. Our analysis identified resection margin status, tumor differentiation, vascular invasion, and adjuvant therapy as independent prognostic factors in this setting.

Surgical margin status is defined by residual tumor: R1 indicates microscopic involvement, whereas R2 indicates gross residual disease. Although both represent incomplete resection, R2 status is associated with a poorer prognosis (median survival 19 vs. 28 months for R1) [8]. HCCA is predominantly adenocarcinoma, most frequently moderately differentiated [23–24]. Tumor differentiation grade is a key pathological correlate of biological behavior and prognosis, with poor differentiation conferring significantly higher metastatic and invasive potential [9–25]. The intricate vasculature of the hepatic hilum facilitates early vascular invasion, a marker of aggressive disease that strongly predicts postoperative recurrence and shorter survival [26–27]. Adjuvant therapy for HCCA is evolving without a unified standard, encompassing chemotherapy, radiotherapy, targeted therapy, immunotherapy, and photodynamic therapy. Postoperative adjuvant treatment has been shown to reduce recurrence risk and improve survival, particularly in patients with positive margins or nodal disease [28–29]. Its strong independent prognostic value in our analysis highlights its critical role in managing residual disease, potentially mitigating the poor prognosis associated with adverse pathological features. It should be noted, however, that adjuvant therapy in our cohort was predominantly gemcitabine-based chemotherapy. The lack of standardization in adjuvant regimens across different institutions and the evolving landscape of targeted therapy and immunotherapy for HCCA mean that the generalizability of this finding requires confirmation in studies with more diverse treatment protocols. Notably, preoperative CA19-9, although significant in univariate analysis, did not retain independent prognostic value in our multivariable model. This suggests that its predictive power is largely mediated by the more direct measures of tumor aggressiveness (vascular invasion) and surgical incompleteness (margin status) captured in our model. In contrast to existing nomograms developed primarily for R0 resection, our model specifically addresses prognosis after R1/R2 resection, filling a notable gap in clinical tools for this challenging patient cohort.

To address this gap, we developed a prognostic nomogram for HCCA patients after R1 or R2 resection based on multivariable Cox regression analysis. The model demonstrated excellent discriminatory ability, with an overall C-index of 0.780. Time-dependent ROC analysis further confirmed its predictive performance, yielding AUCs of 0.904 and 0.822 for 1-year and 2-year survival, respectively. These results indicate that the nomogram can accurately stratify patients into distinct risk categories, especially for predicting near-term (1-year) outcomes. Thus, it provides clinicians with a practical tool for early identification of high-risk patients, enabling more vigilant monitoring and timely intervention.

Evaluation of the calibration curves revealed time-dependent differences in predictive accuracy. The nomogram demonstrated excellent calibration for 1-year survival but overestimated 2-year survival. This finding underscores that the model is primarily optimized for short-term prognostication; its utility for predicting long-term survival (≥ 2 years) is limited, and estimates at later time points should be interpreted with caution. This phenomenon, in which calibration performance declines with longer prediction time, is recognized in prognostic modeling [30–31]. The observed deviation may be attributed to interrelated methodological and biological factors. Methodologically, several aspects warrant consideration. First, patient attrition over time reduces the effective sample size for estimating later survival, affecting calibration precision. Second, the statistical power to precisely estimate time-specific survival probabilities is inherently lower at later time points. Third, although our model incorporates key baseline clinicopathological factors, it may not capture all variables that disproportionately influence longer-term survival, such as the nuanced response to adjuvant therapy. Biologically, the calibration bias may reflect an evolution in the dominant determinants of survival over time. Early survival is likely governed by the initial tumor burden and surgical outcome, which our model captures well. In contrast, later survival may be increasingly influenced by dynamic factors not included in our model, such as the biology of recurrence and subsequent treatment efficacy. This underscores that our model is particularly reliable for near-term prognostication and highlights that later survival is determined by factors extending beyond the baseline surgical-pathological profile.

The clinical application of this nomogram extends to several practical scenarios. First, in the immediate postoperative period, the model can stratify patients into distinct risk categories, enabling tailored adjuvant treatment strategies. High-risk patients (e.g., those with poor differentiation and vascular invasion) may be considered for more aggressive systemic therapy or enrollment in clinical trials evaluating novel agents, while low-risk patients might avoid unnecessary overtreatment. Second, the nomogram can guide the intensity and frequency of postoperative surveillance, with high-risk patients undergoing more frequent imaging to detect early recurrence, whereas low-risk patients may follow a standard surveillance protocol. Third, in preoperative counseling for patients with suspected R1/R2 resection, this model—once externally validated—could theoretically assist surgeons and patients in weighing the potential survival benefit of cytoreductive surgery against the morbidity of the procedure, particularly when compared to palliative drainage alone. For instance, a patient predicted to have very poor post-resection survival might be better served by primary drainage and systemic therapy, avoiding unnecessary surgical trauma. We acknowledge, however, that prospective verification of these clinical application scenarios is needed before widespread implementation.

In conclusion, we present a clinically applicable nomogram for individualized prognosis in patients with HCCA following R1 or R2 resection. It integrates key pathological and therapeutic factors to provide postoperative risk stratification, aiding in the identification of high-risk patients who may benefit from intensified surveillance and prompt multidisciplinary review for adjuvant therapy. The model also facilitates realistic patient counseling. Importantly, it should inform, not replace, clinical judgment, emphasizing that the decision to pursue non-curative surgery necessitates a committed, integrated plan for adjuvant therapy, particularly for patients with high-risk pathological features.

This study has several limitations. First, its retrospective, single-center design is susceptible to potential selection and information biases. Specifically, treatment decisions—including the selection of patients for R1 or R2 resection and the administration of adjuvant therapies—were not randomized and may have been influenced by patient performance status, comorbidities, or surgeon preference, which could confound the observed associations. For instance, the decision to perform R1 or R2 resection and the selection of specific adjuvant therapies were not standardized, which may introduce confounding. Second, although the sample size is justifiable for this rare disease, the absence of an external validation cohort remains a key constraint on the model’s generalizability and stability. As a single-center study from a high-volume tertiary referral center, our findings may reflect specific institutional practices in patient selection, surgical technique, and perioperative care, which could limit their applicability to other settings. This limitation is reflected in the observed overestimation in the 2-year calibration curve, which may be attributed to factors such as long-term patient attrition or unmeasured prognostic variables, including preoperative biliary drainage status and detailed nutritional indices, which were not consistently available in our retrospective dataset. To directly address this critical limitation, our research team has initiated a multi-center collaborative study to prospectively validate the nomogram’s performance across diverse institutional populations. Third, the dichotomization of continuous variables for clinical interpretability may have reduced statistical power and nuanced information. Therefore, further external validation in larger, prospective cohorts is required before this nomogram can be considered for broader clinical application.

## Conclusion

This study developed and validated a prognostic nomogram integrating four independent factors—resection margin status, tumor differentiation, vascular invasion, and adjuvant therapy—for patients with HCCA following R1 or R2 resection. The model demonstrated good discriminative ability and reliable short-term (1-year) predictive accuracy, although its calibration for longer-term (2-year) survival showed overestimation, limiting its utility for extended prognostication. It serves as a practical, evidence-based tool to aid postoperative risk stratification and guide individualized clinical management decisions, particularly in the early postoperative period, for this challenging patient population. Ongoing multi-center validation studies are designed to address this limitation and improve long-term predictive performance.

## Electronic Supplementary Material

Below is the link to the electronic supplementary material.

Supplementary Material 1.

## Data Availability

All data used to support the findings of this study are available on request from the corresponding author.

## Abbreviations

AUC: Area Under the Curve
CA19-9: Carbohydrate antigen 19 − 9
CI: Confidence interval
ECOG: Eastern Cooperative Oncology Group
EPV: Events Per Variable
HCCA: Hilar Cholangiocarcinoma
HR: Hazard Ratio
LASSO: Least Absolute Shrinkage and Selection Operator
ROC: Receiver Operating Characteristic
TBil: Total Bilirubin
