# Supplementary material for: Analysis of prognostic factors for R1/R2 resection in patients with hilar cholangiocarcinoma
Source: BMC Surg. 2026 May 6;26:438. doi: 10.1186/s12893-026-03776-5 (PMC13321434; doi:10.1186/s12893-026-03776-5)
Supplement: Supplementary file 1 — Supplementary Material 1. [file 12893_2026_3776_MOESM1_ESM.doc]

# 安装并加载必要的包

if (!require(readxl)) install.packages("readxl")

if (!require(tidyverse)) install.packages("tidyverse")

if (!require(rms)) install.packages("rms")

if (!require(survival)) install.packages("survival")

if (!require(Hmisc)) install.packages("Hmisc")

library(readxl)

library(tidyverse)

library(rms)

library(survival)

library(Hmisc)

# 1. 读取Excel文件

df <- read_excel("D:\\6shuju.xlsx")

# 2. 数据预处理

df <- df %>%

rename(

time = "time",

status = "status",

margin = "Resection margin status",

grade = "Differentiation grade",

ln_met = "Lymph node metastasis",

vascular = "Vascular invasion",

adjuvant = "Adjuvant therapy"

)

df$time <- as.numeric(df$time)

df$status <- ifelse(df$status %in% c("Dead", "死亡", 1, 2), 1, 0)

df <- df %>%

mutate(

margin = factor(margin, levels = c(0, 1), labels = c("R1", "R2")),

grade = factor(grade, levels = c(3, 2, 1), labels = c("Well", "Moderate", "Poor")),

ln_met = factor(ln_met, levels = c(0, 1), labels = c("No", "Yes")),

vascular = factor(vascular, levels = c(0, 1), labels = c("No", "Yes")),

adjuvant = factor(adjuvant, levels = c(0, 1), labels = c("No", "Yes"))

)

df_clean <- df

# 3. 为变量添加标签

label(df_clean$time) <- "Time (months)"

label(df_clean$status) <- "Status"

label(df_clean$margin) <- "Resection margin status"

label(df_clean$grade) <- "Differentiation grade"

label(df_clean$ln_met) <- "Lymph node metastasis"

label(df_clean$vascular) <- "Vascular invasion"

label(df_clean$adjuvant) <- "Adjuvant therapy"

# 4. 设置rms环境

ddist <- datadist(df_clean)

options(datadist = "ddist")

# 5. 构建COX回归模型

cox_model <- cph(

Surv(time, status) ~ margin + grade + ln_met + vascular + adjuvant,

data = df_clean,

x = TRUE,

y = TRUE,

surv = TRUE,

time.inc = 12

)

# 6. 计算基准生存率

surv_curve <- survest(cox_model)

time_1year <- 12

time_2year <- 24

idx_1year <- which.min(abs(surv_curve$time - time_1year))

idx_2year <- which.min(abs(surv_curve$time - time_2year))

base_surv_1year <- surv_curve$surv[idx_1year]

base_surv_2year <- surv_curve$surv[idx_2year]

# 7. 获取线性预测值的范围，用于调整总分范围

linear_predictor_range <- range(predict(cox_model))

# 缩小线性预测值范围（这里缩小到原始范围的80%）

scale_factor <- 0.8

scaled_range <- linear_predictor_range * scale_factor

# 8. 创建列线图

nom <- nomogram(

cox_model,

fun = list(

function(x) base_surv_1year^x,

function(x) base_surv_2year^x

),

funlabel = c("1-year survival", "2-year survival"),

fun.at = list(

seq(0.05, 0.95, by = 0.05),

seq(0.1, 0.9, by = 0.2)

),

lp = FALSE,

# 关键修改：设置线性预测值的范围，间接控制总分范围

lp.at = seq(scaled_range[1], scaled_range[2], length.out = 10)

)

# 9. 方法一：使用较大的图形设备并调整参数

# 打开一个较大的图形设备

dev.new(width = 30, height = 10) # 宽度14英寸，高度10英寸

# 设置合理的边距

par(mar = c(4, 0, 4, 0)) # 下、左、上、右边距

# 绘制列线图 - 关键修改：移除背景竖虚线

plot(nom,

xfrac = 0.33, # 调整左侧空间比例

cex.var = 0.8, # 变量名称字体大小

cex.axis = 0.65, # 刻度字体大小

col.grid = NA # 将col.grid设置为NA，移除背景竖虚线

)
